# Mastery in Goal Scoring, T-Pattern Detection, and Polar Coordinate Analysis of Motor Skills Used by Lionel Messi and Cristiano Ronaldo

**DOI:** 10.3389/fpsyg.2017.00741

**Published:** 2017-05-12

**Authors:** Marta Castañer, Daniel Barreira, Oleguer Camerino, M. Teresa Anguera, Tiago Fernandes, Raúl Hileno

**Affiliations:** ^1^National Institute of Physical Education of Catalonia, Observation Laboratory in Physical Activity and Sports, University of LleidaLleida, Spain; ^2^Faculty of Sport, Centre of Research, Training, Innovation and Intervention in Sport, University of PortoPorto, Portugal; ^3^Faculty of Psychology, University of BarcelonaBarcelona, Spain

**Keywords:** soccer, goal scoring, motor skills, pattern detection, polar coordinate analysis

## Abstract

Research in soccer has traditionally given more weight to players' technical and tactical skills, but few studies have analyzed the motor skills that underpin specific motor actions. The objective of this study was to investigate the style of play of the world's top soccer players, Cristiano Ronaldo and Lionel Messi, and how they use their motor skills in attacking actions that result in a goal. We used and improved the easy-to-use observation instrument (OSMOS-soccer player) with 9 criteria, each one expanded to build 50 categories. Associations between these categories were investigated by T-pattern detection and polar coordinate analysis. T-pattern analysis detects temporal structures of complex behavioral sequences composed of simpler or directly distinguishable events within specified observation periods (time point series). Polar coordinate analysis involves the application of a complex procedure to provide a vector map of interrelated behaviors obtained from prospective and retrospective sequential analysis. The T-patterns showed that for both players the combined criteria were mainly between the different aspects of motor skills, namely the use of lower limbs, contact with the ball using the outside of the foot, locomotion, body orientation with respect to the opponent goal line, and the criteria of technical actions and the right midfield. Polar coordinate analysis detected significant associations between the same criteria included in the T-patterns as well as the criteria of turning the body, numerical equality with no pressure, and relative numerical superiority.

## Introduction

Soccer performance research is broadly developed and implemented (Ali, 2011), contributing to a rapid and continuous enhancement of players' performance in the last few years (Lago-Ballesteros et al., 2012). Given the complexities and dynamic nature of soccer, observation and measurement processes throughout the design of match analysis systems have made it possible to collect data by embracing technical, behavioral, physical, and tactical factors (Carling et al., 2005).

Due to the high complexity of soccer games, it is known that general research in soccer presents some flaws, such as: (i) lack of context; (ii) missed operational definitions (MacKenzie and Cushion, 2013); and (iii) inability of parameters such as official match statistics and physiological and performance data to provide information for a comprehensive evaluation of the soccer players (Perić et al., 2013). The idiosyncrasies of dynamic systems theory make it possible to overcome these limitations (Glazier and Robins, 2013), entailing that mathematical models of analysis must incorporate a wider range of organismic, environmental, and task constraints (Glazier and Davids, 2009). Specifically, dynamical systems theory plays an important role with its multidisciplinary theoretical framework for sports performance analysis by (i) facilitating the linkage of behaviors to outcomes due to its more process-oriented than product-oriented focus, and (ii) stabilizing the same principles and concepts governing patterns in intra- and inter-individual levels of sports performance (Glazier and Robins, 2013).

There is a general belief that talented people display superior performance in a wide range of activities, such as superior athletic ability and mental abilities (Feltovich et al., 2006). Notwithstanding, to understand sport expertise, multi-scale and multi-disciplinary theoretical descriptions are needed (Araújo et al., 2010). In the domain of team play analysis, McGarry et al. (2002) mention that the main soccer research focuses on tactical and technical factors. Technical analysis includes the testing of key sport skills, including the mechanical aspects of technique, and is concerned with the way the skill is performed in terms of kinetic and kinematic detail of the movement involved (O'Donoghue, 2010). In the perspective of Ali (2011), it becomes useless if the player does not perform the right action at the right time, i.e., when a tactical approach to the players' behavior does not exist. Behavioral specific and representative information is continuously apprehended from the environment by dynamical movement systems, to structure and to adapt functional patterns of play. This sensibility to contextual information regulates the motor system number of biomechanical degrees of freedom, however, more critical than attending each behavior separately, is to form and to develop functional synergies that arise between parts of the body used to achieve movement goals (Davids et al., 2006).

However, with the application of styles of play that incorporate and encourage individual actions and skills, which improve overall game strategies and outcomes (Carling et al., 2008), relevant individual behaviors in soccer, such as goal-scoring, need to be analyzed with regard to motor skills (Castañer et al., 2016b). Although, goal-scoring, the ultimate objective of attacking effectiveness in competition settings, has been extensively used in match performance research (Tenga et al., 2010; Lago-Ballesteros et al., 2012), the objectivity of this research remains insufficient with regard to the motor skills that support goal-scoring patterns (Castañer et al., 2016a).

Indeed, in elite soccer, the use of motor skills has largely been studied from a subjective perspective (Duch et al., 2010), but mastery of these skills (Castañer et al., 2009, 2016a; Wallace and Norton, 2014) is directly linked to motor versatility (Bishop et al., 2013) and consequently to the ability to execute complex intentional actions (Memmert et al., 2013). Motor versatility in both individual and team sports requires the integration of multiple skills (Bishop et al., 2013); it is a particularly important quality in attackers such as strikers and wingers and is closely linked to motor anticipation (Murgia et al., 2014). In fact, the ability to efficiently and effectively execute skilled movement patterns is the most important aspect of soccer performance and players must apply cognitive, perceptual and motor skills to rapidly changing situations (Ali, 2011). These multiple skills are essential to execute soccer moves such as ball control, dribbling, and shots. Motor skills involve axial movements in the form of turns and pivots, spatial orientation of the player's body in relation to the side lines and goal line, and the use of one limb or another (laterality). These movements not only underpin all soccer moves but also contribute to the uniqueness of each player (Castañer et al., 2016a). In addition, most of these movements are interlinked. Laterality (Teixeira et al., 2011; Bishop et al., 2013), for example, refers not only to left-right preference but also to how a player orients his body spatially (Bishop et al., 2013; Loffing et al., 2015). Previous research (Castañer et al., 2016a) has demonstrated that Lionel Messi—a left-footed player whose has achieved some of his best results playing on the right wing—is a good example of laterality. Cristiano Ronaldo does not have the singular characteristic of being left-footed in goal-scoring, but he is also an example of motor skills versatility. This is the main aspect of our research: studying the motor skills that configure the uniqueness of a striker.

Cristiano Ronaldo and Lionel Messi are considered to be the best soccer players who have ever existed. Since 2008, no other player has won the FIFA best player award: Messi has won 5 times and Ronaldo 4 times. In a comparison of Ronaldo's and Messi's goal-scoring in *La Liga* since the 2009–2010 season, Shergold (2016) found that Messi scored 270 goals in 252 matches, playing 21,218 min and taking 953 shots, and Ronaldo scored 270 goals in 247 matches, playing 21,206 min, and taking 1,318 shots. These data show that Messi has a shot conversion rate of 28.77%, compared with 20.03% for Ronaldo. Nevertheless, both players show unusual accuracy as well as uniqueness in motor skills. For instance, Jafari and Smith (2016) hypothesized that Lionel Messi has acquired higher motor skills than most other players, and that this frees up much cognitive capacity. And Hong et al. (2012) describe the “knuckling shot” as one of the characteristics of Ronaldo.

We believe that the above-mentioned attributes, which describe two singular styles of playing soccer, have not been analyzed from an objective, scientific perspective. This sort of analysis is challenging because soccer is a complex game that requires a wide repertoire of individual skills used for the benefit of the team and characterized by constant interactions among technical, tactical, psychological, and physical factors. There are various methods for identifying an expert, for example the retrospective method. Using this method, one can determine who is an expert by looking at how well an outcome or product is received (Chi, 2006). Here we followed Hodges et al. (2006), who assume that tasks are what elucidate the underlying mechanisms that afford consistent expert performance.

Thus, the overall objective of this study was to perform an objective analysis of Lionel Messi's and Cristiano Ronaldo's use of motor skills prior to scoring a goal using two complementary methods: T-pattern analysis and polar coordinate analysis. The methodological aim was to detect temporal structures of behavior underlying the two players' styles by means of T-pattern analysis and, complementarily, to obtain an idea of the behavior in its entirety using polar coordinate analysis, whose powerful data reduction feature facilitates the interpretation of data by means of a vectorial representation of the associations detected between behaviors.

## Methods

Given that our study fulfilled the requisite, established by Anguera (2003), of having perceivable and regular behaviors in a natural setting, we employed systematic observation (Anguera, 1979). The choice of methodology is also justified by the implementation of an *ad-hoc* observation instrument to record, analyze, and interpret the behaviors exhibited by Messi and Ronaldo in the goals analyzed.

Observational methodology offers eight types of observational designs (Blanco-Villaseñor et al., 2003; Sánchez-Algarra and Anguera, 2013; Anguera and Hernández-Mendo, 2014; Portell et al., 2015) that offer different possibilities in terms of the number of participants, the continuity of the recording and the number of criteria observed. These designs have been widely applied in the analysis of individual and team sports (Jonsson et al., 2006; Fernández et al., 2009; Camerino et al., 2012a,b; Lapresa et al., 2013; Castañer et al., 2016a; Tarragó et al., 2016); in the analysis of motor skills in physical activity and sport (Castañer et al., 2009, 2016b) and in mixed methods research in sports (Anguera et al., 2014). We decided to use the N/S/M design, where N refers to nomothetic (focusing on two players), S refers to intersessional follow-up (analyzing specific motor skills and contextual aspects recorded from the beginning to the end of different sequences of numerous matches), and M refers to multidimensional (addressing multiple criteria and responses in the *ad-hoc* observation instrument designed).

Two particularly fitting techniques for the analysis of such complexity are temporal pattern (T-pattern) detection (Casarrubea et al., 2015; Magnusson et al., 2016) and polar coordinate analysis (Sackett, 1980). T-pattern detection has been successfully used in numerous studies to reveal hidden patterns underlying different soccer actions (Anguera and Jonsson, 2003; Jonsson et al., 2006; Fernández et al., 2009; Garzón Echevarría et al., 2011; Lapresa et al., 2013, 2014; Sarmento et al., 2013; Barreira et al., 2014; Escolano-Pérez et al., 2014; Zurloni et al., 2014; Magnusson et al., 2016). Polar coordinate analysis is a powerful data reduction technique that is increasingly being used in studies of team sports (Perea et al., 2012; Robles et al., 2014; Echeazarra et al., 2015; López-López et al., 2015; Morillo-Baro et al., 2015; Sousa et al., 2015; Castañer et al., 2016a; López et al., 2016; Aragón et al., 2017). The technique provides a vectorial representation of the complex network of interrelations between carefully chosen, exhaustive and mutually exclusive defined criteria.

### Participants

A total of 181 goals were analyzed, 83 scored by Lionel Messi and 98 scored by Cristiano Ronaldo (Table 1). The goals were included according to the following criteria:

- Clear observability of the sequence to the goal (Anguera and Hernández-Mendo, 2015);
- Availability of at least two recordings of each sequence from different angles;
- Goals scored in Champions League, *La Liga* and *Copa del Rey* were included;
- Goals had to be from the last three seasons, namely 2013–2014, 2014–2015, and 2015–2016;
- Opponent quality criterion: Champions League is known as the most elite Union of European Football Associations (UEFA) competition, so all goals scored in this league were considered; in *La Liga* and *Copa del Rey*, the criteria of Bradley et al. (2013) were followed, i.e., only the goals scored against non-successful clubs—the last four classified at the end of each season—were not included;
- Goals resulting directly from set pieces, including the rebound of a penalty, were not considered;
- Goals scored in regular time were included.

**Table 1:** **Goals scored by Lionel Messi and Cristiano Ronaldo considered**.

**Competition**	**Season**	**Lionel Messi**	**Cristiano Ronaldo**	**Total**
Champions League	2013–2014	5	13	18
	2014–2015	10	7	17
	2015–2016	5	12	17
	Total	20	32	52
*La Liga*	2013–2014	15	17	32
	2014–2015	26	26	52
	2015–2016	11	22	33
	Total	52	65	117
*Copa del Rey*	2013–2014	4	0	4
	2014–2015	3	1	4
	2015–2016	4	0	4
	Total	11	1	12
	Total	83	98	181

Our study can thus be considered case-oriented (Sandelowski, 1996; Yin, 2014). The goals were analyzed using public television footage, in compliance with the ethical principles of the Declaration of Helsinki.

### Materials

#### Observational instrument

The *ad-hoc* observation instrument OSMOS-soccer player (Castañer et al., 2016a) was used with a minimal optimization of criteria. Specifically, the criterion Number of Opponents was replaced by Centre of the Game, adapted from Barreira et al. (2012, 2014, 2015), and the criterion Stability, which includes jumps, was merged with the Turn and Pivot Direction criteria. The instrument (see Table 2) comprised nine criteria: (1) Body Part (part of the body that the player uses to make contact with the ball); (2) Foot Contact Zone (part of the foot used to touch the ball); (3) Body Orientation (angle of the chest with respect to the side line or goal line); (4) Stability (turn direction, right vs. left; pivot foot, right vs. left; and elevation of the body); (5) Locomotion (number of steps between touches of the ball); (6) Action (common soccer technical actions); (7) Centre of the Game (number of players on both teams interacting during the striker's action); (8) Side (position of the player on the pitch); and (9) Zone (area where the player moves). Each criterion was expanded to build an exhaustive and mutually exclusive observation system that included, in total, 50 categories.

**Table 2:** **OSMOS-soccer player (Observation system for motor skills in soccer)**.

**Criterion**	**Category**	**Code**	**Description**
1. Body part	Left foot	LF	Player touches the ball with left foot
	Right foot	RF	Player touches the ball with right foot
	Left leg	LL	Player touches the ball with left leg (not including foot)
	Right leg	RL	Player touches the ball with right leg (not including foot)
	Chest	CH	Player touches the ball with chest
	Back	BA	Player touches the ball with back
	Head	HD	Player touches the ball with head
2. Foot contact zone	Tip	TI	Player touches the ball with tip of foot
	Outside	OU	Player touches the ball with outside of foot
	Inside	ID	Player touches the ball with inside of foot
	Heel	HL	Player touches the ball with heel
	Sole	SO	Player touches the ball with sole
	Instep	IT	Player touches the ball with instep
	Non-observable	NON	No clear contact zone between player and ball
3. Body orientation with respect to rival goal line	Facing goal	FG	Player's chest facing rival goal line
	Facing right	OR	Player's chest facing right side line
	Back to goal	BT	Player's back facing rival goal line
	Facing left	OL	Player's chest facing left side line
4. Stability	Right turn	RT	Player makes a full or half turn to the right (vertical axis)
	Left turn	LT	Player makes a full or half turn to the left (vertical axis)
	Right foot pivot	RFP	Player pivots to the right on right foot
	Left foot pivot	LFP	Player pivots to the left on left foot
	Jump	JUM	Elevation of the body
5. Locomotion	One	ONE	Player takes one step without touching the ball
	Two	TWO	Player takes two steps without touching the ball
	Three	THR	Player takes three steps without touching the ball
	Four	FOU	Player takes four steps without touching the ball
	Five	FIV	Player takes five steps without touching the ball
	More	MOR	Player takes more than five steps without touching the ball
6. Technical actions	Control	CT	Player gains control of the ball following diverse actions
	Dribbling	CD	Player dribbles the ball
	Shot	SH	Player shoots
	Feint (shot)	SHF	Player pretends to shoot.
	Feint (pass)	PAF	Player pretends to pass
	Feint (change of dir.)	DIF	Player tricks a defender by changing direction
	Volley	VO	Player makes contact with the ball before it touches the ground
7. Centre of the game	Relative numerical inferiority	PR	Attacking team has one or two influent players less than the opponent in the centre of the game
	Absolute numerical inferiority	PA	Attacking team has at least less three or more influent players in relation with the opponent in the centre of the game
	Numerical equality with pressure	PE	Attacking team has the same number of players than the opponent in the centre of the game. The ball carrier has his back oriented to the opponent's goal and an opponent is marking from behind
	Numerical equality with no pressure	NPE	Attacking team has the same number of players than the opponent in the centre of the game. The ball carrier has his chest oriented to the opponent's goal, with conditions to progress into the pitch offensive zones
	Relative numerical superiority	NPR	Attacking has one or two influent players more than the opponent in the centre of the game
	Absolute numerical superiority	NPA	Attacking team has three or more influent players than the opponent in the centre of the game
8. Side	Right wing	RW	Part of the pitch between the right side line and the right midfield
	Right midfield	CR	Part of the pitch between the left midfield and the right side line
	Left midfield	CL	Part of the pitch between the right midfield and the left side line
	Left wing	LW	Part of the pitch between the left side line and the left midfield
9. Zones	Ultraoffensive 1	UOO	Between the goal line and the front of the goal area
	Ultraoffensive 2	UOT	Between the front of the goal area and the penalty box
	Offensive	OFF	Between the front of the penalty box and the half-way line (excl. centre circle)
	Central	CEN	Centre circle

#### Recording instrument

Goal-scoring sequences were coded using LINCE (v.1.2.1) (Gabín et al., 2012). This software program was also used for the data quality check.

#### Data analysis software

Two programs were used: (a) Theme software package (Magnusson et al., 2016) for T-pattern detection; (b) HOISAN v.1.6.3.2 (Hernández-Mendo et al., 2012, 2014) for the polar coordinate analysis.

### Procedure

Goal-scoring sequences were analyzed from the moment the player receives the last pass to the moment he scores a goal. After appropriate training in the use of OSMOS-soccer player, two expert observers—an expert soccer analyst and a motor skills expert—recorded 30% of the total goals included for each player (Table 3). Intra- and inter-observer reliability was calculated in LINCE, before the full data set was coded, using a preliminary dataset of 55 and 30 goal-scoring sequences, respectively. The goals used to calculate data quality were from the 2012 to 2013 season and therefore were not included in the final sample. The resulting kappa statistic was 0.95 for inter-observer and 0.98 for intra-observer analysis, which guarantees the interpretative rigor of the coding process.

**Table 3:** **Reliability: sample and values per player and for both players**.

	**Lionel Messi**			**Cristiano Ronaldo**			**Total**		
	***n***	**%^a^**	**Kappa**	***n***	**%^a^**	**Kappa**	***n***	**%^a^**	**Kappa**
Inter-observer	25	30	0.95	30	31	0.94	55	30	0.95
Intra-observer	15	18	0.98	15	15	0.97	30	17	0.98

*n = number of goals*.

a *Percentage of goals with regard to the final sample (see Table 1) for both players*.

### Data analysis

#### T-pattern detection

T-pattern detection is a relevant data analysis technique in systematic observation (Anguera and Hernández-Mendo, 2015) and the THEME software is a powerful research tool for obtaining T-patterns. This software makes it possible to explore behavioral structures in detail by revealing stronger connections between successive recorded behaviors in goals than would be expected by chance. The critical interval is the key concept that makes it possible to delimit the admissible temporal distances between successive identical or similar occurrences in order to consider the existence of a temporal pattern. Obtaining T-patterns is a procedure of great importance for theoretical and empirical purposes, and deriving their algorithm has involved the development of powerful new analytic techniques based on probability theory and, more specifically, on binomial distribution (Magnusson, 2000). Three criteria were applied to guarantee that any T-patterns detected were not due to random events: (a) presence of a given T-pattern in at least 25% of all sequences, (b) significance level of 0.005, and (c) redundancy reduction setting of 90% for occurrences of similar T-patterns. As Magnusson states, the idea of T-pattern analysis is to detect repeated behavioral patterns that are invisible to unaided observers. The temporal structure of complex behavioral sequences is composed of simpler or directly distinguishable event-types (Magnusson et al., 2016). Each T-data set subject to analysis consists of series of behaviors coded as occurrence times (beginning and end points) within specified observation periods (time point series; Magnusson, 1996).

**Figure 1:**
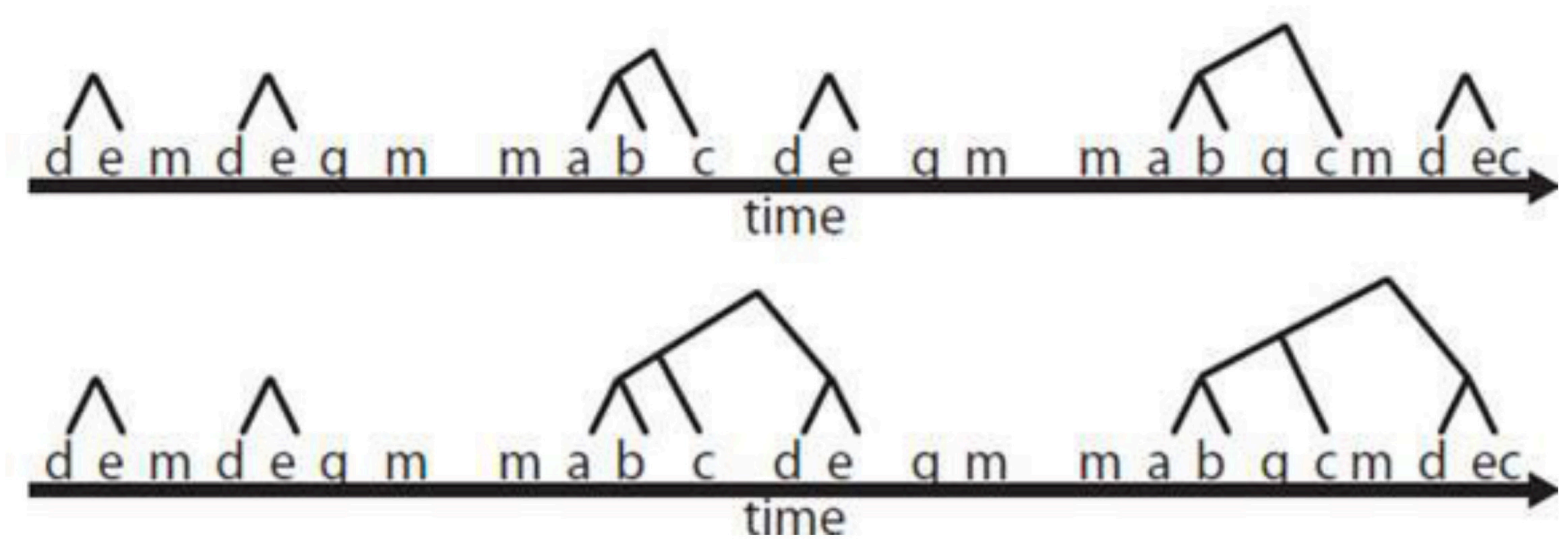
**Formation of a T-pattern from a simple T-pattern (first line) to more complex ones, such as the T-pattern at the bottom (Castañer et al., 2009, p. 859)**.

More specifically, the following explanation, used in several studies, allows a clear understanding of how T-pattern detection works. For instance, in a given observation period, two repeated actions, A and B, either in this same order or simultaneously, form a minimal T-pattern (AB) if they are found more often than would be expected by chance, and if, assuming the null hypothesis of independent distributions for A and B, they are separated by approximately the same distance (time). Instances of A and B separated by this approximate distance constitute an (AB) T-pattern and their occurrence times are added to the original data. More complex T-patterns consisting of simple, already-detected patterns are subsequently added through a bottom-up detection procedure. Pairs or series of patterns can thus be detected, for example (((AB)C)(DE)) (see Figure 1).

The THEME software compares all patterns and retains only the most complete ones. Although, only a limited range of basic unit sizes is relevant in any study, T-patterns are, in principle, scale-independent as any basic time unit can be used. Thus, it would be fruitful in the study of Messi's and Ronaldo's goal-scoring.

#### Polar coordinate analysis

The structure of polar coordinate analysis, a technique of sequential analysis (Bakeman, 1978), is based on the complementarity between two analytical perspectives: prospective and retrospective. Polar coordinate analysis involves the detection of significant associations between *focal behavior* (the behavior of interest) and *conditional behaviors* (the other behaviors analyzed).

To define a focal behavior, it is first necessary to conduct the prospective analysis, which, depending on the aims of the study, is believed to generate or trigger a series of connections with other categories, known as conditional behaviors. The retrospective, or “backward” perspective, which incorporates what Anguera (1997) referred to as the concept of “genuine retrospectivity,” reveals significant associations between the focal behavior and behaviors that occur before this behavior.

The technique of polar coordinate analysis can be applied to a series of values that are independent of each other, which is the case of adjusted residuals, whether prospective or retrospective, as they are calculated separately for each lag. Standardized Z statistics derived from adjusted residuals (Bakeman, 1978, 1991) corresponding to both prospective and retrospective lags are needed to compute prospective and retrospective Zsum statistics. These values, which can be positive or negative and are located in one of four quadrants, are then used to build maps showing the relationships between a focal behavior (Gorospe and Anguera, 2000; or a criterion behavior, as it is known in lag sequential analysis) and one or more conditional behaviors. Polar coordinate analysis involves the application of a complex procedure to provide a vector map of interrelated behaviors. The same number of prospective and retrospective lags is analyzed in each case. Prospective lags show which conditional behaviors precede the given behavior, while retrospective lags show which behaviors follow it.

As mentioned above, polar coordinate analysis merges the prospective and retrospective approaches to achieve a powerful reduction of data through the calculation of the *Z*_*sum*_ statistic $\left(\frac{\Sigma z}{\sqrt{n}}\right)$ described by Cochran (1954) and later developed by Sackett (1980). In both the prospective approach (Z_sum_P) and the retrospective approach (Z_sum_R), calculations are based on the frequency of the given behavior, *n*, and a series of mutually independent *z*-values for each lag. Each of these values is obtained by applying the binomial test to compute conditional probabilities (based on the number of codes recorded for each goal sequence) and unconditional probabilities (due to random effects). The length of each vector is obtained from ${\sqrt{{\left(Z_{\text{sum}}\text{P}\right)}^{2}+\left(Z_{\text{sum}}R\right)}}^{2}$, while its angle is calculated by dividing the retrospective *Z*_*sum*_ arcsine by the radius (φ = arcsine of Y/radius). Prospective and retrospective *Z*_*sum*_ values (lags 1–5 and lags −1 to −5, respectively) can carry a positive or negative sign; these signs determine in which quadrant the resulting vectors (behaviors) are placed. To illustrate the results, a map with four quadrants indicates the relationship (inhibitory vs. excitatory) between the focal and conditional behaviors. Thus, each quadrant reveals the following relationships:

Quadrant I (++). The given and conditional behaviors are mutually excitatory.
Quadrant II (− +). The given behavior is inhibitory and the conditional behavior is excitatory.
Quadrant III (− −). The given and conditional behaviors are mutually inhibitory.
Quadrant IV (+ −). The given behavior is excitatory and the conditional behavior is inhibitory.

As in previous research (Castañer et al., 2016a), Figure 2 gives a graphical explanation of how to interpret the associations between given and conditional behaviors depending on the quadrant.

**Figure 2:**
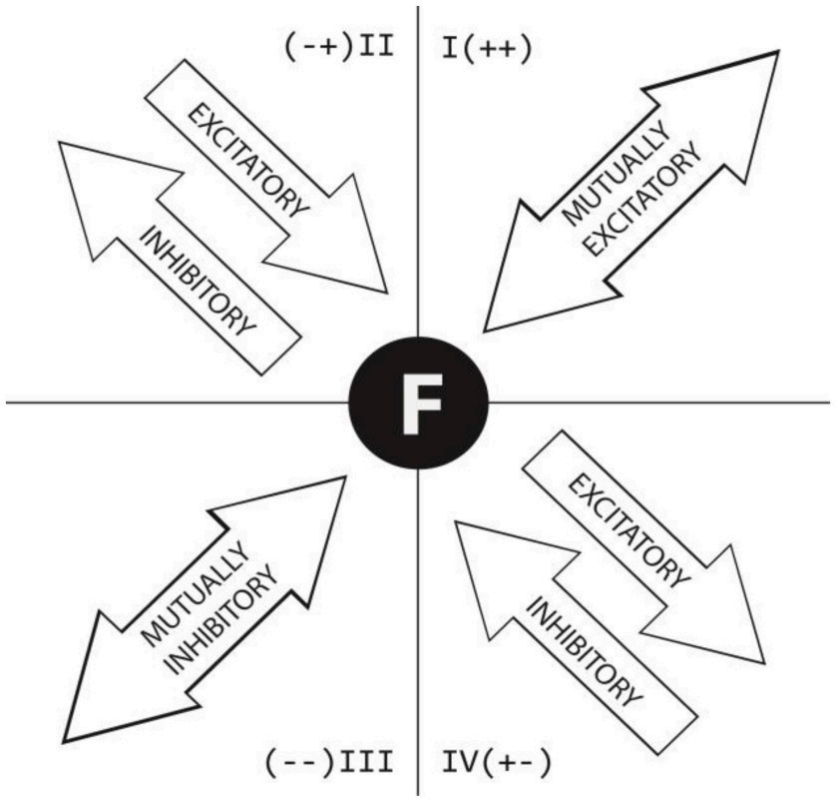
**Graphic depiction of relationships between conditional and given behaviors in polar coordinate maps according to the quadrant in which the vector is located (Castañer et al., 2016a, p. 5.)**.

In each polar coordinate map, the focal behavior is placed in the middle and, depending on the quadrant in which the conditional behavior is placed, the angle of the vector is transformed as follows: quadrant I (0 < φ <90) = φ; quadrant II (90 < φ <180) = 180 – φ; quadrant III (180 < φ <270) = 180 + φ; quadrant IV (270° < φ <360°) = 360° − φ.

The HOISAN v1.6.3.2 software was used to calculate the prospective and retrospective adjusted residuals and the length and angle of the vectors and to produce a graphical representation of the results obtained.

## Results

### T-pattern detection

**Figure 3:**
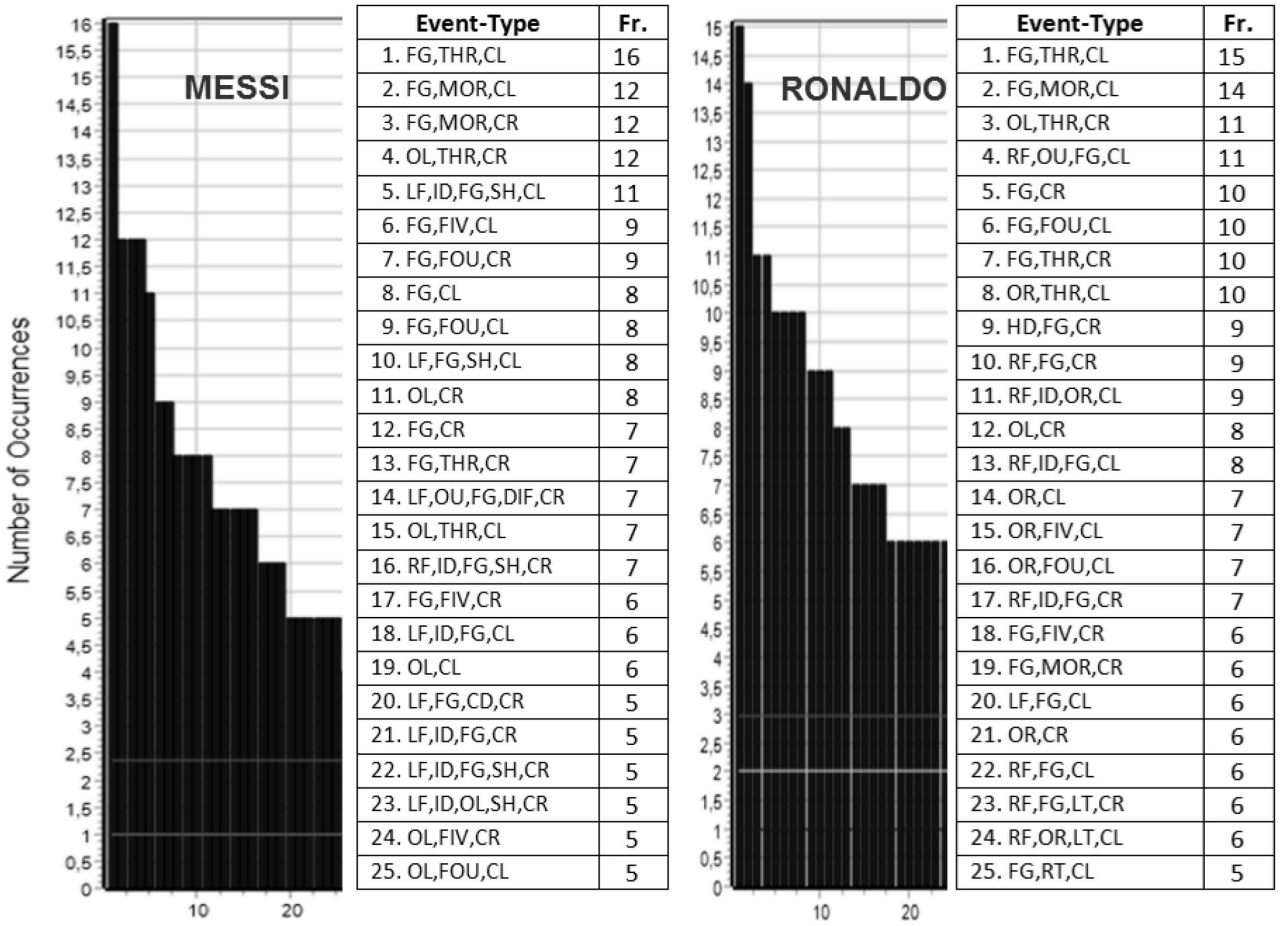
**Messi and Ronaldo event-type frequency chart**.

T-pattern detection was performed using the free THEME software. Firstly, we explored the frequency of events and event sequences (Figure 3). The box in Figure 3 shows the first 25 event-types with more than 2 occurrences (Messi in the left chart and Ronaldo in the right chart).

The most frequent event-types for both players were a total of nine configurations of codes. These were: facing goal, three steps between touches in the left midfield (FG,THR,CL) (Messi, *n* = 16; Ronaldo, *n* = 15); facing goal, more than five steps in the left midfield (FG,MOR,CL) (Messi, *n* = 12; Ronaldo, *n* = 14); facing goal, more than five steps in the right midfield (FG,MOR,CR) (Messi, *n* = 12; Ronaldo, *n* = 6); left orientation of the body with respect to the rival goal line, three steps in the right midfield (OL,THR,CR) (Messi, *n* = 12; Ronaldo, *n* = 11); facing goal, four steps between touches in the left midfield (FG,FOU,CL) (Messi, *n* = 8; Ronaldo, *n* = 10); left orientation of the body in the right midfield (OL,CR) (Messi, *n* = 8; Ronaldo, *n* = 8); facing goal in the right midfield (FG,CR) (Messi, *n* = 7; Ronaldo *n* = 10); facing goal, five steps in the right midfield (FG,FIV,CR) (Messi, *n* = 6, Ronaldo, *n* = 6); and facing goal, three steps in the right midfield (FG,THR,CR) (Messi, *n* = 7, Ronaldo, *n* = 10).

Other detectable aspects shown on the frequency chart are the fact that Messi used his left foot (LF) in 8 configurations and his right foot (RF) in 1 configuration. Ronaldo used his right foot (RF) in 8 configurations and his left foot (LF) did not appear in any configuration of codes. Messi used the left body orientation (OL) with respect to the rival goal line in 7 configurations of codes and the right body orientation (OR) did not appear in any configuration. Ronaldo used the right body orientation (OR) with respect to the rival goal in 7 configurations and the left body orientation (OL) appeared in 2 configurations.

**Figure 4:**
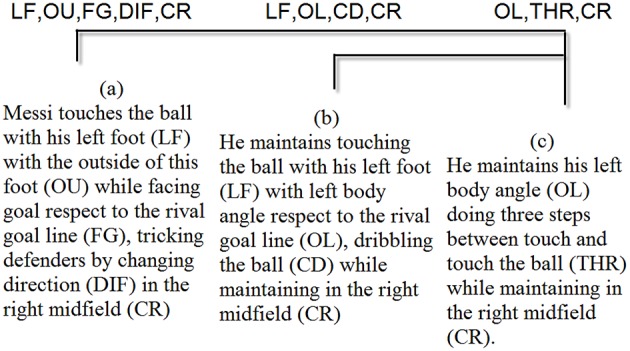
**T-pattern shows that (a) Messi touches the ball with his left foot (LF) with the outside of this foot (OU) while facing the rival goal line (FG), tricking defenders by changing direction (DIF) in the right midfield (CR); then, (b) he continues to touch the ball with his left foot (LF) with a left body angle with respect to the rival goal line (OL) and dribbles the ball (CD) while remaining in the right midfield (CR); and then, (c) he maintains his left body angle (OL), takes three steps between touches of the ball (THR) and remains in the right midfield (CR)**.

**Figure 5:**
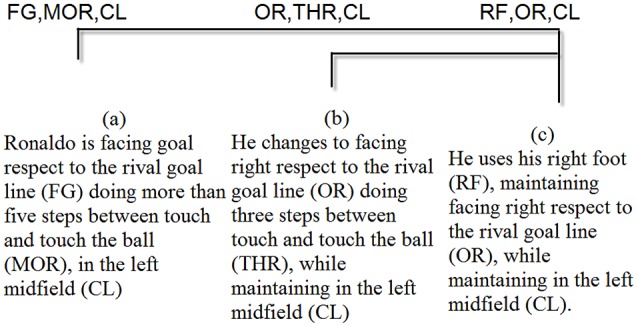
**T-pattern shows that (a) Ronaldo is facing the rival goal line (FG), takes more than five steps between touches of the ball (MOR) in the left midfield (CL); then, (b) he changes to facing right with respect to the rival goal line (OR) and takes three steps between touches of the ball (THR) while remaining in the left midfield (CL); and (c) he uses his right foot (RF), remaining facing right with respect to the rival goal line (OR), while remaining in the left midfield (CL)**.

Obtaining T-patterns allows us to show a broad view of the main sequences that the two players use in the process of goal-scoring. As any basic time unit can be used, the T-pattern technique selects the range of basic unit sizes that are relevant in any study. For this study, the categories that appeared in the T-patterns were: Body Part, Foot Contact Zone, Body Orientation, Action and Side. Figures 4, 5 show the most complete T-patterns detected for Messi and Ronaldo, respectively.

### Polar coordinate analysis

**Figure 6:**
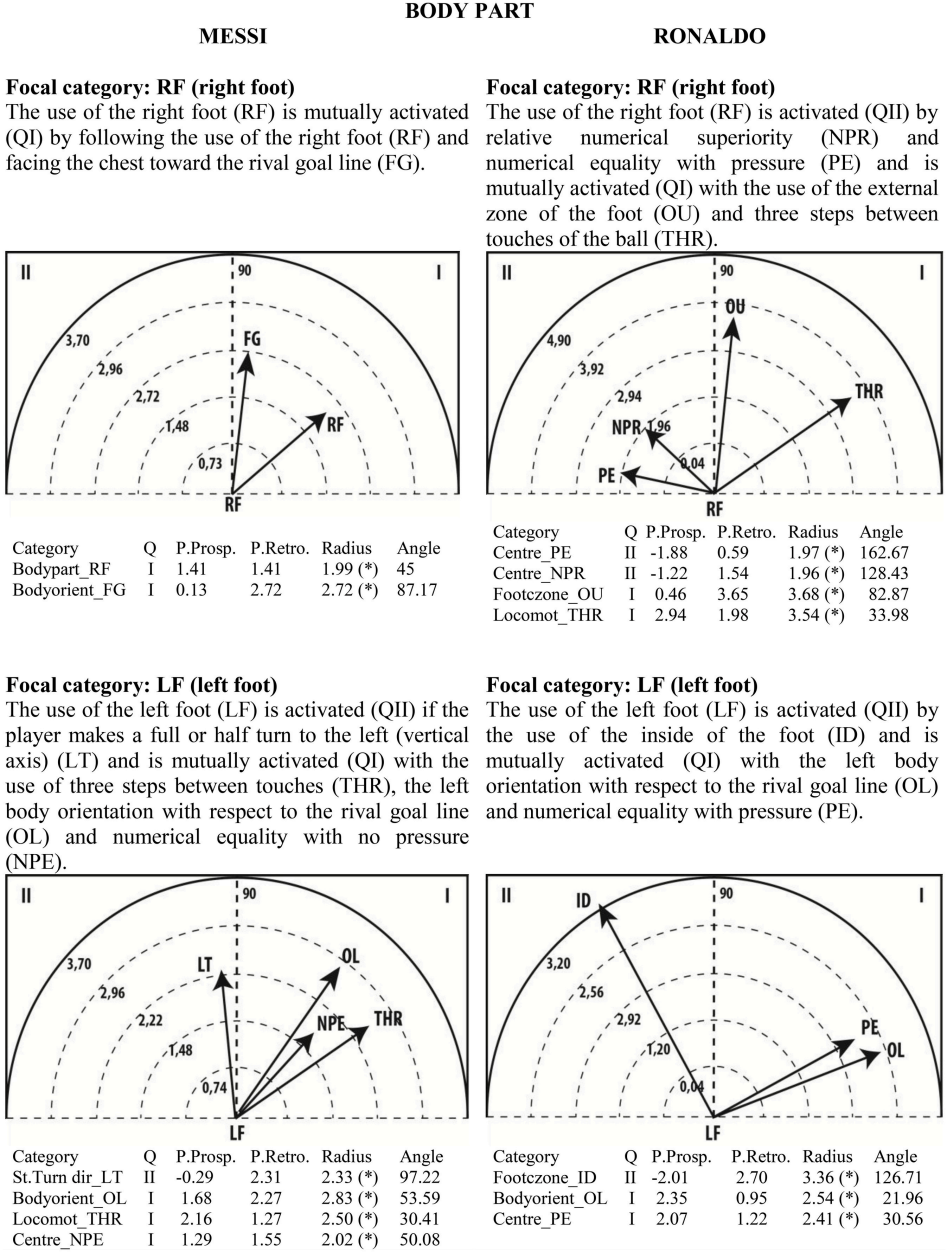
**Maps of polar coordinate analysis for the Body Part criterion**.

**Figure 7:**
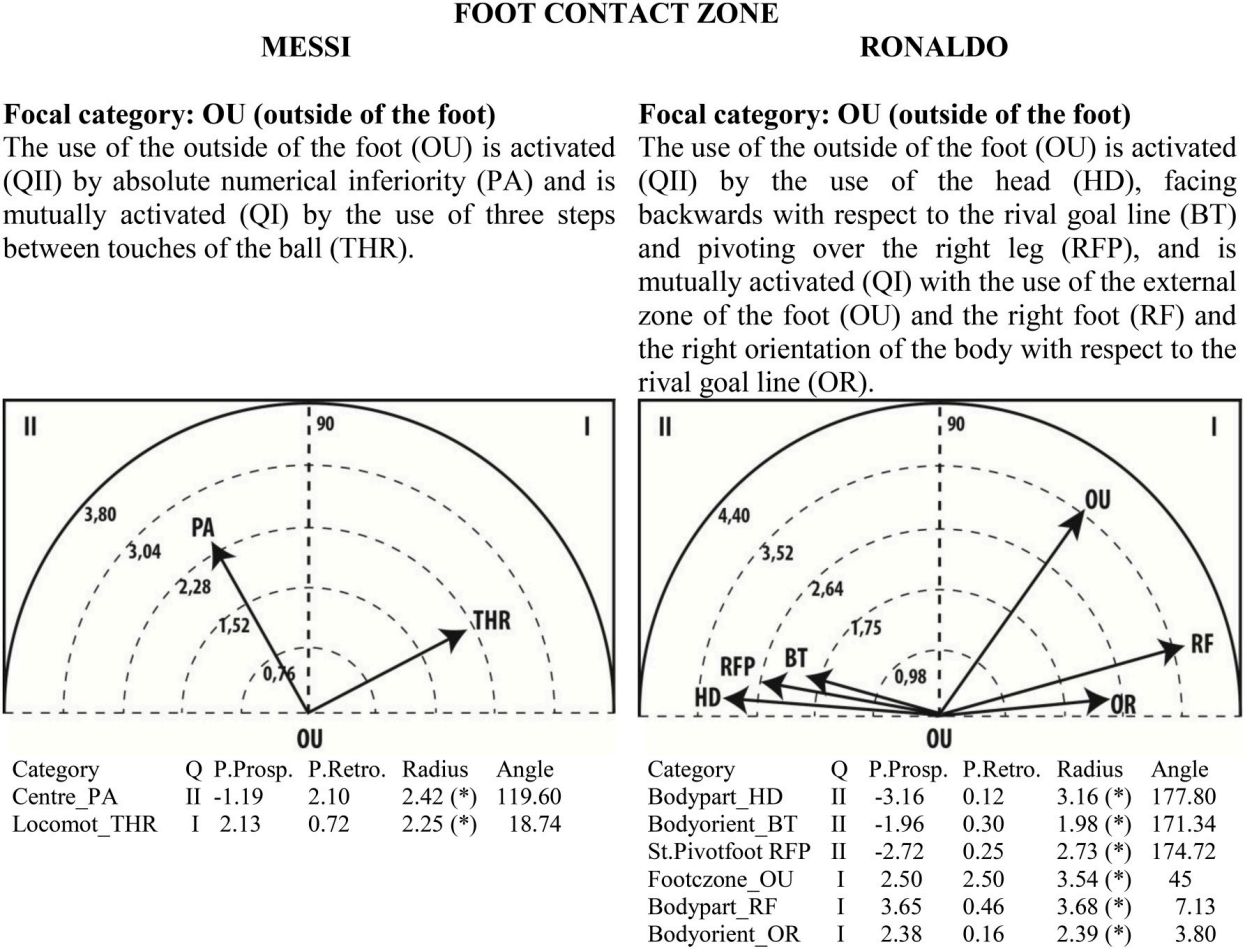
**Maps of polar coordinate analysis for the Foot Contact Zone criterion**.

**Figure 8:**
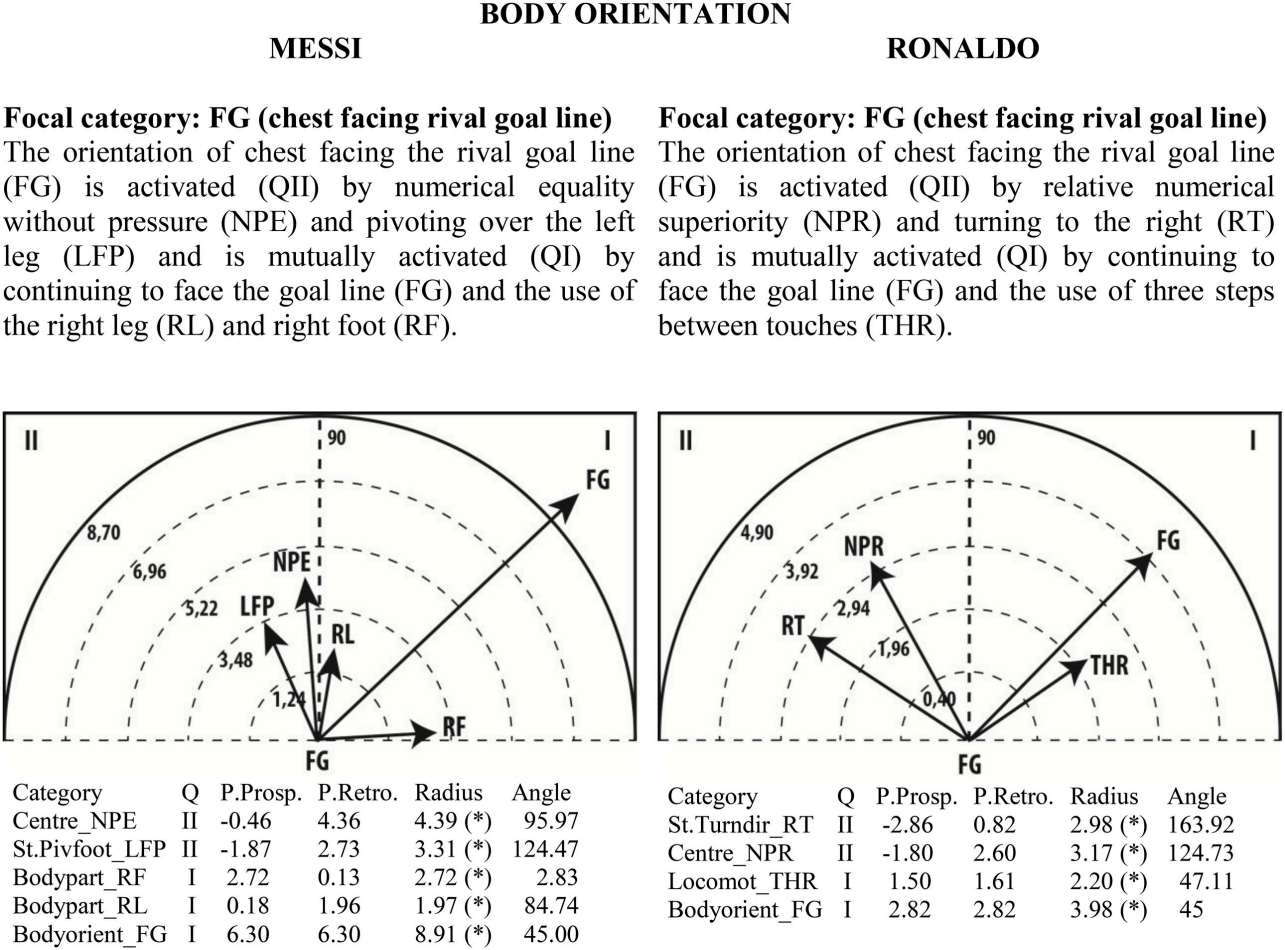
**Maps of polar coordinate analysis for the Body Orientation criterion**.

**Figure 9:**
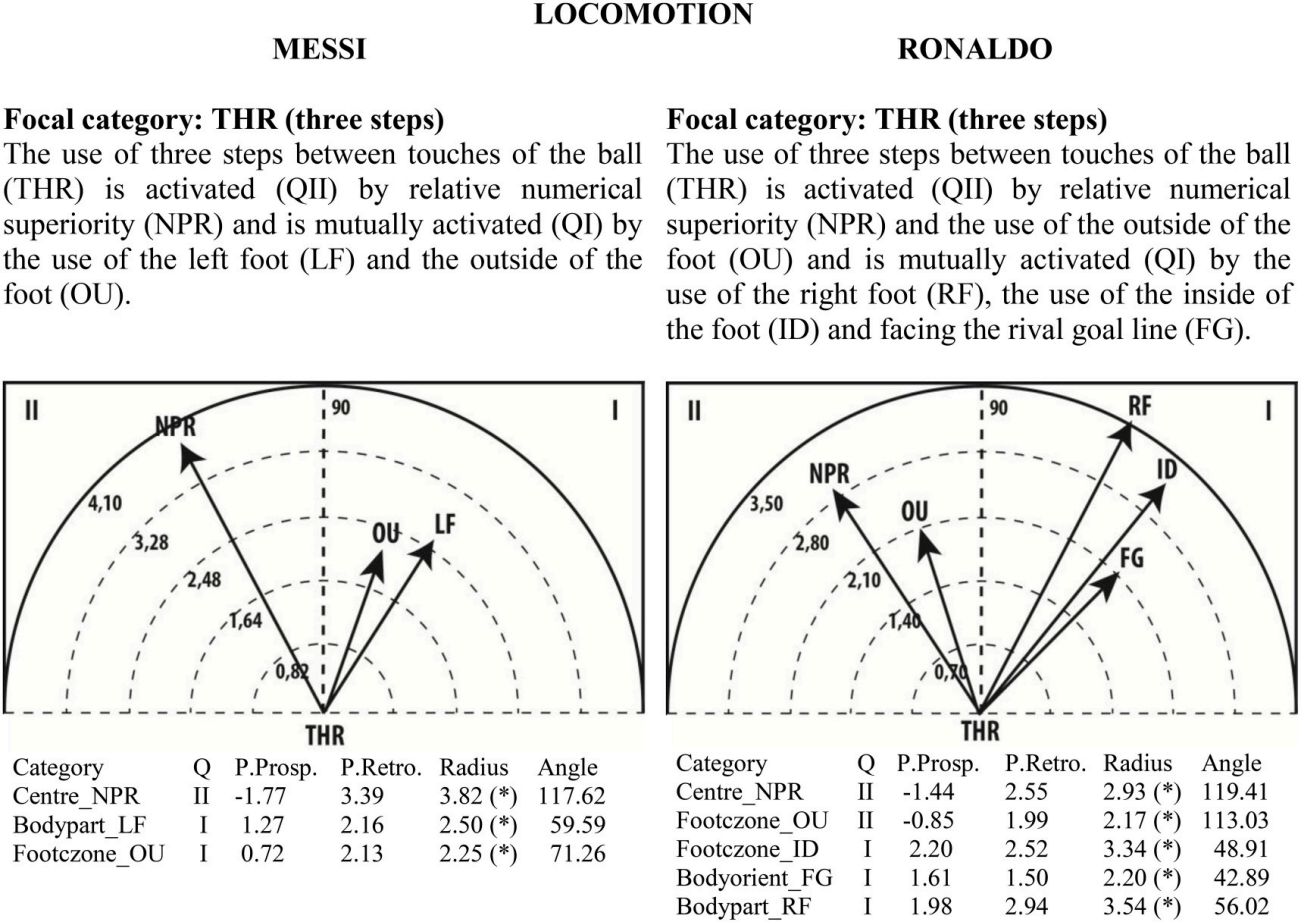
**Maps of polar coordinate analysis for the Locomotion criterion**.

**Figure 10:**
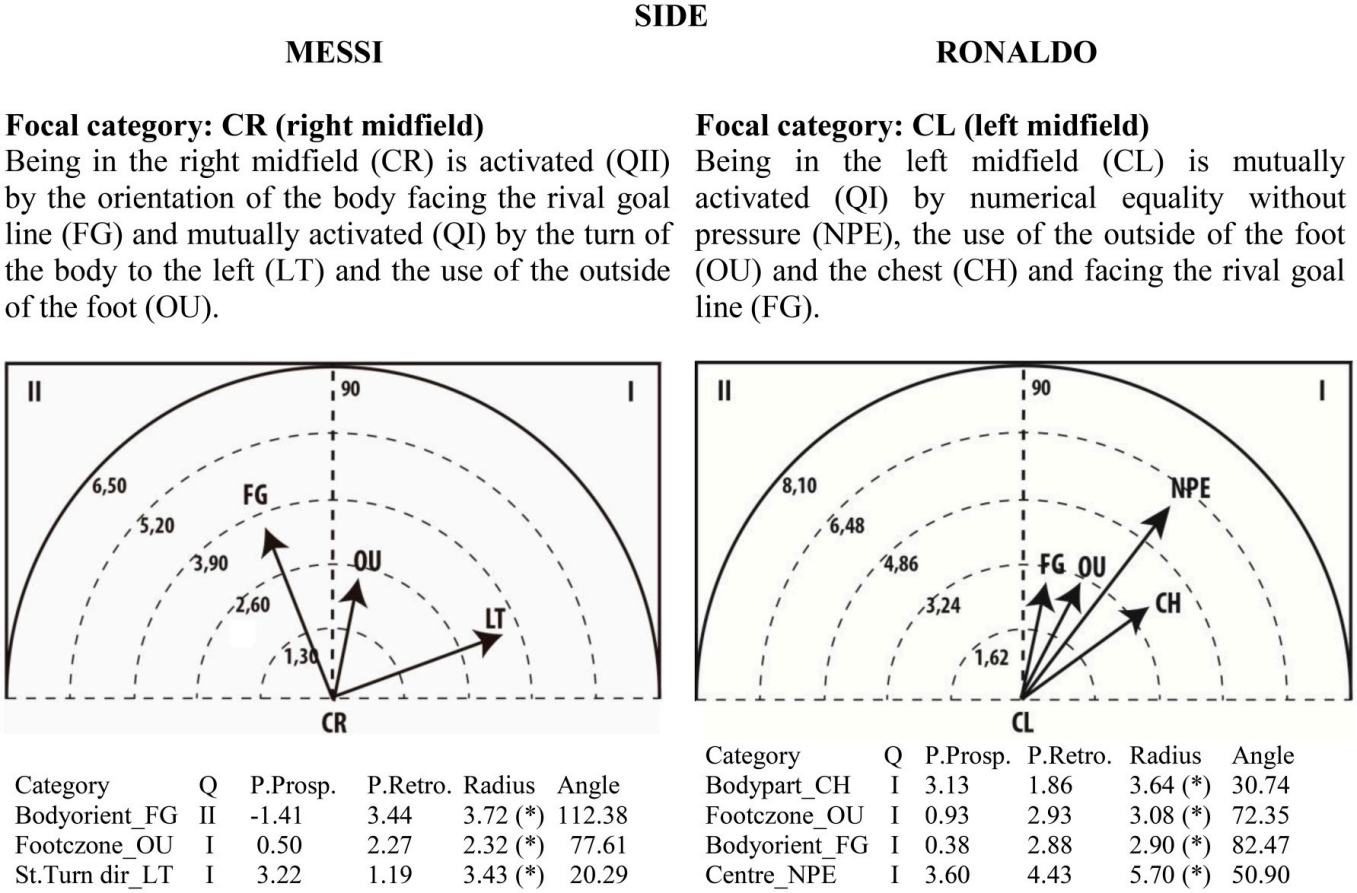
**Maps of polar coordinate analysis for the Side criterion**.

**Figure 11:**
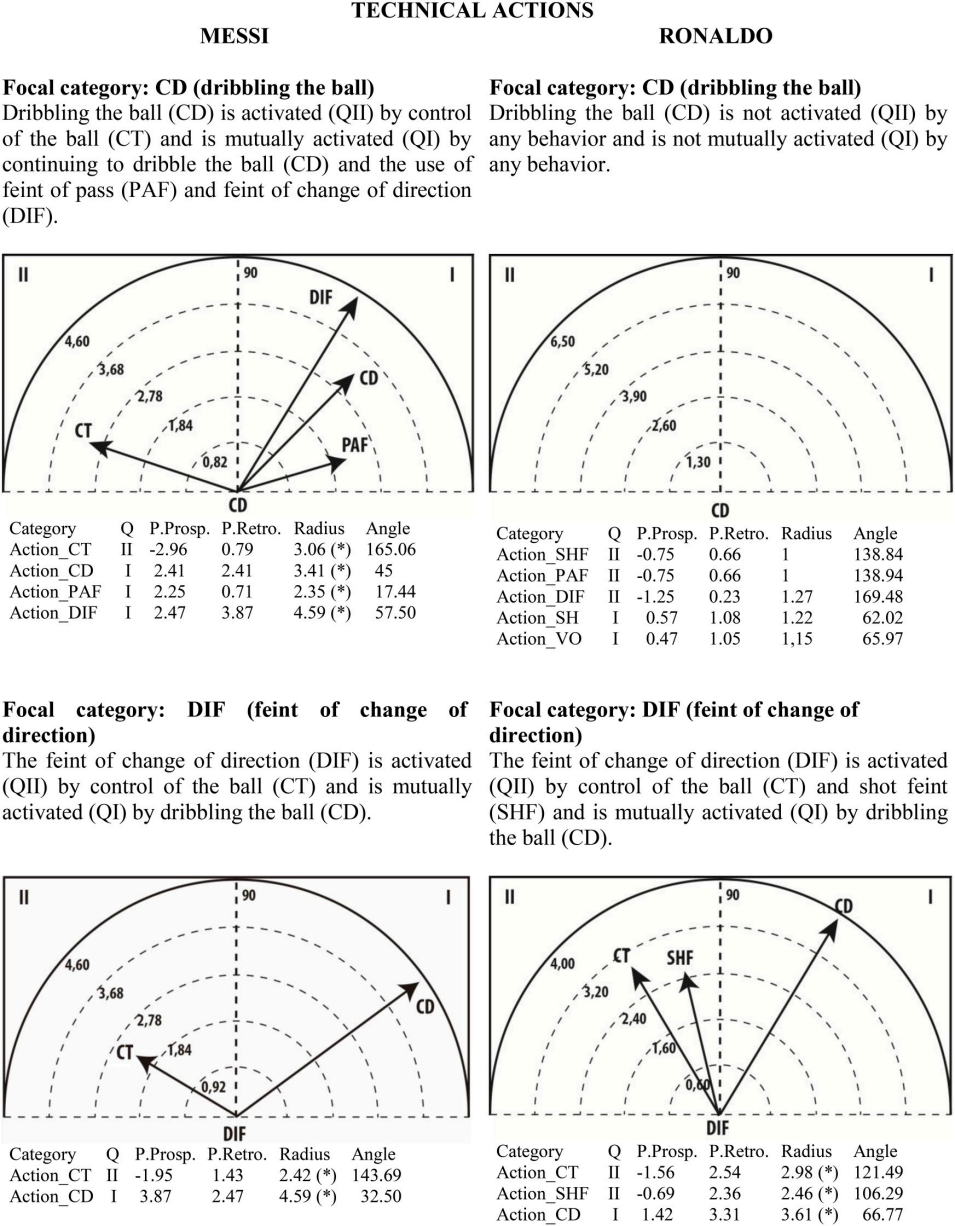
**Maps of polar coordinate analysis for the Technical Actions criterion**.

**Figure 12:**
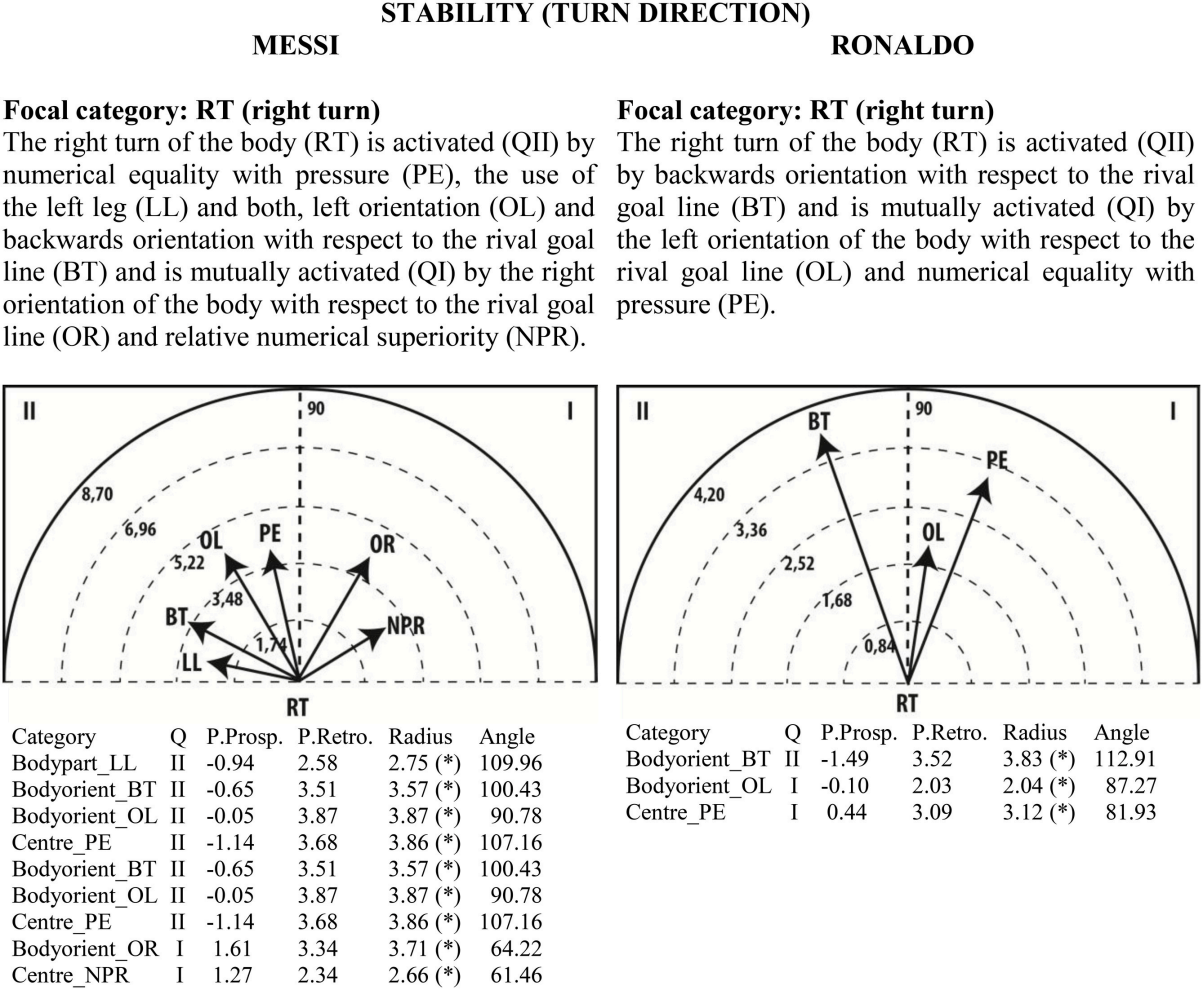
**Maps of polar coordinate analysis for the Stability (turn direction) criterion**.

**Figure 13:**
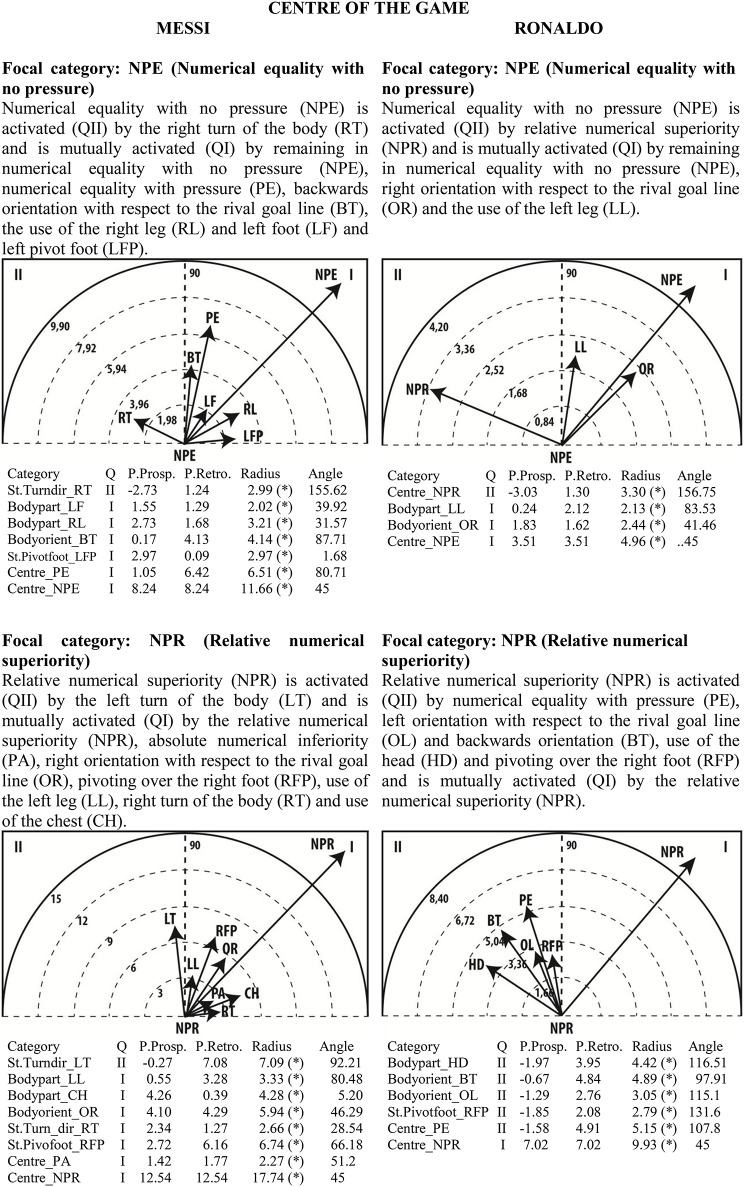
**Maps of polar coordinate analysis for the Centre of the Game criterion**.

Given the clear understanding of the associations between focal and conditional behaviors provided by Figure 2, we selected quadrant II (QII), which contains the conditional categories that activate the focal category, and quadrant I (QI), which contains the categories that have mutual activation with the focal category. The maps in Figures 6–13 show both quadrants with the length and angle of the vectors with a length of >1.96 (*p* <0.05) for the behaviors that show statistically significant associations (activation).

Figures 6–13 show the results of polar coordinate analysis for Messi and Ronaldo concerning the categories in quadrant II (QII), which activate the focal category, and those in quadrant I (QI), which are mutually activated by the focal category. We include below each semicircle map the table of values statistically obtained. Firstly, we expose the categories that appear in the T-patterns corresponding to the following criteria: Body Part, Foot Contact Zone, Body Orientation, Locomotion and Side. Complementarily, we offer the polar coordinate analysis for the criteria Stability (turn direction) and Centre of the Game, which have also shown statistically significant activation between them.

## Discussion

The objective of this study was to perform an objective analysis of Lionel Messi's and Cristiano Ronaldo's use of motor skills prior to scoring a goal using the complementary methods of T-pattern analysis and polar coordinate analysis.

The structure of the Discussion Section is as follows. First, we comment on the polar coordinate analysis results following the order of criteria in the OSMOS-soccer player instrument. Second, we comment on the findings of the T-patterns analysis. Each section ends with clues about how experts can understand the findings in order to improve their professional work.

### Body contact with the ball

Polar coordinate maps show great differences between the two players with regard to the use of the right foot. While there are no behaviors by Messi that activate the use of the right foot, Ronaldo's use of the right foot is promoted in situations of relative numerical superiority and numerical equality with pressure and is mutually activated by the use of the external zone of the foot and taking three steps between touches of the ball. In contrast, maps for the use of the left foot show more mutual activations between behaviors for Messi and fewer for Ronaldo. Moreover, Messi's use of the left foot and the left body orientation with respect to the rival goal line is induced by turning the body to the left. The use of three steps between touches and numerical equality with no pressure seem to be behaviors mutually activated with the use of the left foot. These maps reinforce the notion that Ronaldo and Messi tend to use a preferred foot—right and left, respectively—in situations without high pressure and while dribbling to create advantage in attacking zones and in one-on-one situations. Moreover, the findings of Castañer et al. (2016a) related to the contralateral dominance of Messi's body orientation are corroborated. Our results also verify the findings of Carey et al. (2001), which showed that players mostly used the preferred foot when performing set pieces and the technical actions of first touching, passing, dribbling, and tackling. Furthermore, Carey et al. (2001) highlighted that players were more asymmetrical for set pieces than for the dynamic phases of the game.

The use of the inside of the foot activates Ronaldo's left foot use and this is mutually activated with numerical equality with pressure (PE) and, like Messi, the left body orientation with respect to the rival goal line.

Likewise, T-pattern analysis clearly shows the predominant use of the left foot by Messi and the right foot by Ronaldo. Despite the great differences between the two players in terms of the use of the right and left foot, the polar coordinate maps and frequency chart also show the players' versatility and adaptability in using both feet with other behaviors when necessary. Carey et al. (2001) found that very few players used both feet with equal frequency, but on those rare occasions they showed similar performance with the preferred and non-preferred feet. We therefore advise experts that the successful use of both feet, notwithstanding with different frequency, thus evidencing versatility, is an indicator of expertise in soccer and as such could be included as a coaching task in order to develop symmetrical use of both feet during dynamic interaction with the ball.

### Foot contact zone

For Messi, the use of the outside of the foot is activated by absolute numerical inferiority and activates the use of three steps between touches of the ball. The use of the outside of the foot by Ronaldo is activated by the use of the head, facing backward with respect to the rival goal line and pivoting over the right leg, and is mutually activated with the use of the external zone of the foot and the right foot, as well as the right orientation of the body with respect to the rival goal line (OR). This finding fits with the logic of soccer play: players usually use the exterior part of the foot to run with the ball faster.

### Body orientation with respect to the goal line

As the main task of strikers is goal-scoring, it is not surprising that both players use the body orientation of facing the rival goal line in interaction with other behaviors. We emphasize that contexts with no pressure induce both players to face the goal—the context of numerical equality without pressure in Messi's case and relative numerical superiority in Ronaldo's case. This result shows that expert players have great anticipation capacities, corroborating the finding of Ericsson (2003) that experts seem to be better at catching early relevant indicators of the specific task. In our study, Messi and Ronaldo seem to create positional advantages in relation to the rival goal by using their attention abilities to better anticipate the outcomes of their actions and the actions of opponents (Afonso et al., 2012). So, in direct relation with the ball, they have already prepared conditions to have higher success in attacking situations.

Messi's goal-facing orientation is mutually activated mainly with remaining facing the goal line, with the use of the right leg and with the use of the right foot. As Messi is left-footed, the use of the right foot and leg while facing the rival goal line does not seem to us to be a paradox but rather an indication of his versatility in the use of contralateral inferior limbs, as the values of the polar coordinate analysis are very low. These findings are consistent with the findings of previous research (Castañer et al., 2016a).

### Locomotion

Both polar coordinate analysis and T-pattern detection detected the locomotion behavior of taking three steps between touches of the ball. In both players, this behavior is activated by relative numerical superiority. Also, we found that Messi and Ronaldo use the outside part of the foot, a category that was activated in Ronaldo's map and mutually activated in Messi's map. These results could be interpreted to mean that in no-pressure conditions of play the exterior part of the foot is the part used most often in dribbling because with this ability the players create more speed conditions in order to gain an advantage in space in relation to their opponents. The singular contralateral use of the feet of both players is again reinforced by these maps, which show the mutual activation of taking three steps and the use of the left foot in Messi's case and the right foot in Ronaldo's case.

### Side

The right and left midfield are the categories of the Side criterion identified by polar coordinate analysis and T-pattern detection. T-patterns show clearly the difference between the two players in relation to the main uses of the midfield (the right midfield by Messi and the left midfield by Ronaldo). The presence of Messi in the right midfield is activated by the body orientation facing the rival goal line and is mutually activated by turning the body to the left and the use of the outside of the foot. The presence of Ronaldo in the left midfield is activated by numerical equality without pressure, the use of the outside of the foot, the use of the chest and facing the rival goal line. These results corroborate statistics presented by InStat Scout software about Messi's and Ronaldo's patterns of play with regard to where the players touch the ball throughout the matches: 84% of Messi's touches occur in the right wing, 8% in the mid-offensive zone, and 8% in the central attacking zones; Ronaldo touches the ball mostly in the left wing (57%), followed closely by the central attacking zones (42%). These data show that Ronaldo tends to play in interior zones of the field more frequently than Messi.

### Technical actions

T-pattern detection and the frequency chart show more use of dribbling and feint of change of direction in Messi's goal-scoring than in Ronaldo's. Polar coordinate maps also show non-statistically significant activation between dribbling and other behaviors. Contrarily, Messi's dribbling is activated by control of the ball and is mutually activated with continuing to dribble the ball, the use of feint of pass and the use of feint of change of direction. The T-pattern detection also reinforces this behavior (Figure 4): Messi touches the ball with the outside part of his left foot while facing the rival goal line. To do this, Messi tricks defenders by changing direction in the right midfield and then continuing to touch the ball with his left foot with a left orientation of the body with respect to the rival goal line; then, he continues dribbling the ball while remaining in the right midfield. We therefore conclude that Messi tends to create a great diversity of individual attacking situations, a result that corroborates the conclusion of Serrado (2015): that Messi is the world's most unpredictable player. Morris (2014), studying Messi between 2010 and 2014, reported that he has 50% efficacy in dribbling and tries to perform feints on average 8 times per game. He also showed that Messi was the most successful player in assists and goals scored, having the best goals/assists ratio with 1.30 goals and 0.40 assists per game. In the same period of analysis, in passing situations Messi was the striker with the most passes performed (11,120), 84% of them successfully. Of these passes, 47% were completed to attacking zones, with 450 through balls, 30 of them permitting a goal (Morris, 2014).

The maps also show that feint of change of direction is more similar in the two players. This behavior is activated by the control of the ball and is mutually activated with dribbling. For Ronaldo, it is also mutually activated by the shot feint, which corroborates the notion that Ronaldo was the top shooter in the 2010–2014 period, with 1,018 shots performed (Morris, 2014).

### Stability (turn direction)

The maps show for Messi that the right turn of the body is activated by the use of the left leg and being oriented backwards with respect to the rival goal line and is mutually activated with the right body orientation with respect to the rival goal line and relative numerical superiority. This finding reinforces again the contralateral actions of stasis and precision of the laterality uses of the limbs (Teixeira et al., 2011). For Messi and also for Ronaldo, the map shows that the right turn of the body is activated by facing backwards with respect to the rival goal line and is mutually activated with the left body orientation with respect to the rival goal line and numerical equality with pressure for Ronaldo and right body orientation for Messi. Along similar lines, Castañer et al. (2016a) reported that the right turn of the body showed that Messi's goal-scoring was directly related to the use of the left leg because he remains steady over his right leg in order to turn the body, allowing the left leg to perform precise actions.

### Centre of the game

The most relevant aspect that can be seen in the behavior of numerical equality with no pressure is that in both players it is mutually activated by the continuation of numerical equality with no pressure. We conclude that expert players frequently create conditions, in time and space, to play in no-pressure conditions, in this case in goal-scoring situations. Anticipation is generally considered a hallmark of experts, so it should be considered on the basis of the specific tasks and contexts with knowledge of their advantages and disadvantages (Gold and Shadlen, 2007). Messi and Ronaldo, as the most expert goal scorers, seem to create better conditions to apply shooting technique.

### Conclusions and future lines of study

The objective of this study was to describe objectively the singular goal-scoring style of the world's top soccer players, Cristiano Ronaldo and Lionel Messi. Observational methodology allows sports scientists to obtain objective data to complement subjective judgments of soccer players' motor skill use. We used the OSMOS-soccer player observational system (Castañer et al., 2016a), applying six criteria related to the players' motor skills and three criteria related to tactics and contextual aspects. This instrument is a good fit for our study because we consider that going deeply into the motor skills that players use could be of interest to soccer studies, which are traditionally more focused on the tactical and technical analysis of teams. The combination of two powerful observational techniques, namely T-pattern detection and polar coordinate analysis, allowed us to describe the “mosaic” of motor skills and contextual aspects that make up the singular style of play of Messi and Ronaldo, two of the best soccer players in the world in the early twenty-first century.

Our findings permit us to conclude that Messi and Ronaldo exhibit motor skills that allow them to create varied conditions for goal-scoring. The cumulative use of these abilities, over the course of matches and seasons, allows them to win the top awards in soccer. Here we detail our most important results:

- The creation of no-pressure conditions in goal-scoring shows that both players use attention abilities to better anticipate the outcomes of their motor actions and the actions of their opponents, resulting in higher success in attacking.
- The players exhibited symmetry in the use of both feet with success. However, in conditions of goal-scoring, i.e., with no pressure in the centre of the game, both players mostly used the foot with better laterality precision.
- Ronaldo and Messi mainly use the exterior part of the foot to dribble faster in order to create advantage in attacking zones and in one-on-one situations.
- The players exhibited great versatility in the use of a vast variety of motor skills and technical actions in goal-scoring contexts. Messi is considered an unpredictable player in his goal-scoring actions and Ronaldo an accurate shooter with more recurring patterns.

As for the practical implications of this study, in the Discussion Section we indicated the findings that could be of interest for coaches and for further related studies. Overall, coaches may use these findings for task manipulation related to skill acquisition and improvement of goal-scoring efficacy. Also, studies of this type could be useful for establishing defensive strategies against these specific players. Thus, it would be interesting for future research to consider others types of contexts or outcomes, for example World Cup competition and shots off target, respectively, to better discriminate between the motor ability patterns of successful and unsuccessful performances.

## Author contributions

MC developed the project and supervised the design of the study and the drafting of the manuscript. DB was responsible for the review of the literature and the drafting of the manuscript. OC was responsible for the T-pattern detection, data collection/handling and the critical revision of the content. MA performed the polar coordinate analysis and the method section. TF collected and codified the data. RH supervised the drafting of the manuscript. All authors approved the final, submitted version of the manuscript.

## Funding

We gratefully acknowledge the support of INEFC (National Institute of Physical Education of Catalonia) and the support of two Spanish government projects (Ministerio de Economía y Competitividad): (1) La actividad física y el deporte como potenciadores del estilo de vida saludable: Evaluación del comportamiento deportivo desde metodologías no intrusivas (Grant number DEP2015-66069-P); (2) Avances metodológicos y tecnológicos en el estudio observacional del comportamiento deportivo (PSI2015-71947-REDP); and the support of the Generalitat de Catalunya Research Group, Grup de Recerca i Innovació en Dissenys (GRID). Tecnología i aplicació multimedia i digital als dissenys observacionals (Grant number 2014 SGR 971).

### Conflict of interest statement

The authors declare that the research was conducted in the absence of any commercial or financial relationships that could be construed as a potential conflict of interest.
